# Learning how to recover from stress: Results from an internet-based randomized controlled pilot trial

**DOI:** 10.1016/j.invent.2023.100681

**Published:** 2023-10-17

**Authors:** Robert Persson Asplund, Fernanda Carvallo, Hanna Christensson, Elin Videsäter, Annakarin Häggman, Brjánn Ljótsson, Per Carlbring, Gerhard Andersson

**Affiliations:** a Linköping University, Linköping, Sweden; b Karolinska Institute, Sweden; c Stockholm University, Sweden

**Keywords:** Recovery, Stress, Work, Internet-based, Intervention, Prevention

## Abstract

• One of the first trials examining the efficacy of a brief preventive recovery training program in a sample of distressed employees.
• Preliminary results suggest that employees across a wide range of professions could learn to recover, reduce stress, and improve quality-of-life.
• This type of accessible and brief recovery intervention might shape the future of workplace stress prevention, however, more research is needed.

## Abstract (sv)

**Introduction:**

This randomized, controlled pilot trial evaluated the efficacy of a brief internet-based recovery training intervention targeting a clinical sample of distressed employees.

**Method:**

A sample of 69 employees with elevated symptoms of stress were assigned randomly to a five-week guided recovery training intervention (iRTP, n = 35) or a wait-list control (WLC, n = 34). The study was conducted in Sweden and participants enrolled via an open recruitment strategy. Self-report data were collected pre- and post-intervention, then six and 12 months after the intervention. The primary outcome measure was the Recovery Experience Questionnaire (REQ. The secondary outcome measures gauged other relevant mental and work-related health outcomes. Participants in the wait-list control group received access to iRTP after the six-month follow-up.

**Results:**

Compared with the controls, participants in the intervention group showed a significant and large overall improvement on the primary outcome REQ (*d* = 0.93), and small to moderate effects on the secondary outcomes including, perceived stress (*d* = 0.48), anxiety (*d* = 0.49), quality of life (*d* = 0.47), and work ability (*d* = 0.47) during post-assessment. No significant differences were found at any time point regarding burnout, exhaustion, depression, physical exercise, work experience, or sickness absences.

**Conclusion:**

This pilot trial is one of the first to examine a brief recovery training program's efficacy, suggesting that employees across a wide range of professions could learn how to recover from elevated stress symptoms. This type of accessible and brief recovery intervention could potentially prevent and reduce the negative effects of stress, as well as improve recovery and quality of life. However, more research is needed with larger samples before further conclusions can be drawn.

**Trial Registration:**

The study was registered at Clinical Trials (clinicaltrials.gov) number NCT05220592.

## 1 Introduction

### 1.1 Theoretical background

Stress has increasingly been recognized as a significant health issue, especially within the working population (Atroszko et al., 2020; Eurofound, 2017). In addition to well-known health implications (e.g., coronary artery disease, metabolic syndrome, and mental health disorders) work-related stress has been associated with work disability, absence from work, and loss of productivity (Eddy et al., 2017; Hassard et al., 2018; Joyce et al., 2016; Litwiller et al., 2017; Madsen et al., 2017).

Recovery from work is especially important in preventing and reducing the negative effects of stress, as well as preserving health, well-being, and performance (evidence from meta-analyses, e.g., Bennett et al., 2018; Steed et al., 2021; Wendsche and Lohmann-Haislah, 2016). According to (Sonnentag et al., 2017) *recovery* refers to the restoration processes during which a person's stress level returns to its pre-stress level. Geurts and Sonnentag (2006) asserted that a distinction exists between recovery as a *process* and recovery as an *outcome*. Recovery as a process refers to the activities and experiences that elicit change in stress indicators. Recovery as an outcome captures a person's psychological or physiological state reached after a recovery period (e.g., at the end of a workday). Confirmatory factor analysis has proposed four distinct experiences in recovering from work processes: psychological detachment; relaxation; mastery; and control (Sonnentag and Fritz, 2007). *Psychological detachment* refers to the subjective experience of mentally leaving work and stressors and associated worries during non-work time. *Relaxation* describes the experience of feeling mentally and physically relaxed. *Mastery* comes from the experiences of achievement, for instance, when engaging in a new hobby. *Control*, captures the experience of deciding oneself about what, when, and how to do during non-work time (Sonnentag et al., 2017). Studies confirm that these experiences are positively related to well-being indicators (Fritz et al., 2010; Siltaloppi et al., 2009). Consequently, Sonnentag and Fritz (2007) developed and validated a questionnaire corresponding to these experiences, the Recovery Experience Questionnaire. Sonnentag et al. (2017) concluded in an overview article that psychological detachment and relaxation exhibit the most consistent evidence.

Although extensive research has been devoted to stress intervention programs and evidence from systematic reviews and meta-analyses suggests that these are effective in reducing stress within the working population (Bhui et al., 2012; Miguel et al., 2023; Richardson and Rothstein, 2008). Most stress interventions have concentrated on reducing participants' cognitive, emotional, and behavioral symptoms when coping with elevated stress or an established stress-related disorder. However, promising results have been reported from prospective studies on psychological detachment interventions and recovery training programs (Almén et al., 2020; Ebert et al., 2015; Hahn et al., 2011; Karabinski et al., 2021). These interventions have the advantage of mainly focusing on promoting stress-reducing recovery techniques, such as detachment from work. Given their focus, RTPs could potentially be effective in stress prevention. For example, a pioneering study by Hahn et al. (2011) reported positive effects of a recovery training program (RTP) on outcomes related to recovery experiences, self-efficacy, sleep quality, perceived stress, and negative affect. No effects were found on a measure of emotional exhaustion. However, the study by Hahn et al. (2011) was a quasi-experimental evaluation, including healthy participants, and covering a four-week follow-up period. Hence, we still lack knowledge from controlled trials (RCT) in clinical samples and on the long-term benefits of RTPs for distressed employees. Since the study by Hahn et al. (2011), a growing number of studies have been conducted mainly focusing on psychological detachment from work interventions. A recent meta-analysis (Karabinski et al., 2021) covering 30 studies (*N* = 3725) found significant positive effects (*d* = 0.36) on interventions for improving psychological detachment from work. Additionally, the same analysis concluded that interventions with longer durations and higher dosages were more effective than shorter and lower dosage interventions, and interventions were more effective among participants with initial health or recovery-related impairments.

Despite the evidence on the efficacy of stress management interventions (Bhui et al., 2012; Miguel et al., 2023) and promising results from studies on psychological detachment and recovery training interventions (e.g., Hahn et al., 2011; Karabinski et al., 2021), a majority of individuals suffering from stress and other mental health-related disorders, remain untreated (Ebert et al., 2019). This calls for further development and evaluation of interventions that are accessible and has the potential in preventing chronic stress in the working population.

The internet has the potential to disseminate interventions broadly, and a growing body of literature has demonstrated the efficacy of internet-based stress interventions (Heber et al., 2017; Phillips et al., 2019; Svärdman et al., 2022). Studies also suggest that internet-based interventions can have effects on both stress and work-related outcomes, such as absenteeism (Persson Asplund et al., 2023; Stratton et al., 2021), exert long-term stress reduction (Lindsäter et al., 2018; Persson Asplund et al., 2018; Wiencke et al., 2016), be cost-effective (Ebert et al., 2018; Lindsäter et al., 2019), and have positive effects on health and well-being in both work and private life (Persson Asplund et al., 2019). Notwithstanding the promising results, few controlled studies have examined the efficacy of internet-based interventions focusing on promoting recovery and restorative behavior among distresses employees (e.g., Behrendt et al., 2020; Ebert et al., 2015; Thiart et al., 2015). In a comparable study by Thiart et al. (2015) a guided internet-based intervention was evaluated among 128 teachers with insomnia. The internet-based intervention consisted of six sessions including methods from cognitive behavioral therapy for insomnia, as well as techniques targeted at reducing rumination and promoting recreational activities. Compared to a waitlist control group, the between-group analysis found moderate to large effects on recovery experiences (control Cohen's *d* = 0.34–0.39, mastery *d* = 0–0.05, psychological detachment *d* = 0.64–0.77, relaxation *d* = 0.42–0.72), recreational activities (*d* = 0.58–0.34), and insomnia (*d* = 1.45–1.43), eight weeks and six months after the intervention (Thiart et al., 2015). However, the study only included teachers and, as the authors pointed out, it would be interesting to examine whether these results extended other health issues, e.g. stress, and to a wider range of professions.

### 1.2 Aims

This pilot trial was planned and designed with the overall aim of providing information regarding content, recruitment strategies, and retention rates, and providing preliminary evidence of the efficacy potential of a brief internet-based recovery training program targeting a clinical sample of employees experiencing elevated symptoms of stress. In the present study, the term stress-related disorders refers to non-traumatic stress disorders, including adjustment disorder and other stress reactions, triggered by identifiable stressors (e.g., divorce or job loss). We expected that the internet-based recovery program would produce greater improvements in recovery experiences (primary outcome) compared with a wait-list control group. We also expected that the intervention group would differ with regard to important health-related (perceived stress, burnout, exhaustion, depression, alcohol consumption, and quality of life) and work-related (work experience, work ability, sickness absences) outcomes. Finally, we investigated whether the initially achieved changes in the intervention group would remain stable at the six- and 12-month follow-ups.

## 2 Method

### 2.1 Design

**Fig. 1:**
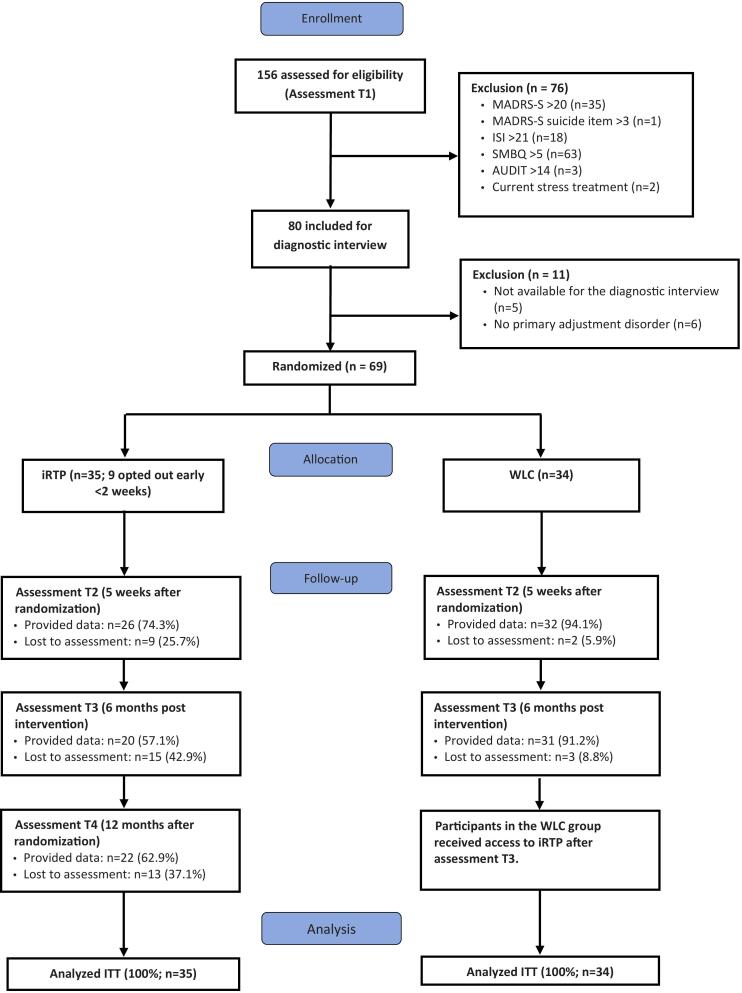
The flow of study participants. iRTP = Internet-based recovery training program; WLC = Waitlist control.

In this randomized, controlled pilot trial, an internet-based recovery training program (iRTP) was compared with a wait-list control group (WLC). The study followed Consolidated Standards of Reporting Trials (CONSORT) guidelines (Schulz et al., 2010) and was conducted between February 2018 and May 2019. Since this was one of the first iRTP, the pilot trial was planned and designed with the overall aim of providing information regarding content, recruitment strategies, and retention rates, and providing preliminary evidence of efficacy potential. Hence, no power calculation and estimates of sample size were conducted. Self-report outcome measures were collected at pre- and post-treatment (five weeks) and six- and 12-month follow-ups (Fig. 1). Participants who met the study criteria and provided informed consent were allocated randomly by an independent researcher using an online random generator (www.randomizer.org). All participants and coaches were randomized in a 1:1 proportion, allocating n = 35 to the iRTP group and n = 34 to the WLC group. The Ethical Committee of Linköping University, Sweden, approved all procedures used in the study (Reference No. 2016/11-31). The study was registered retrospectively at Clinical Trials (clinicaltrials.gov) reference number NCT05220592. The study design and trial were planned and executed according to the ethics approval and clinical trials registry.

### 2.2 Procedure

The study was delivered via an encrypted online treatment platform, *iTerapi*, hosted by Linköping University (Vlaescu et al., 2016). Participants were recruited from the general public through ads, articles in regional and national newspapers, and social media, using an open recruitment strategy. Detailed information and an application form for the study were presented on the project's homepage (www.istress.se). After initial registration, potential participants received an ID number and were asked to (i) provide written informed consent, (ii) complete online screening questionnaires (see Measures), and (iii) participate in a diagnostic telephone interview. Following the interviews, the selected participants were randomized. Participants in the iRTP group received access to the program immediately after randomization, and participants in the wait-list control group received access to iRTP after the six-month follow-up.

### 2.3 Inclusion and exclusion criteria

All participants were volunteers. To be eligible for the study, each participant had to fulfill the following criteria: (i) at least 18 years old; (ii) fluent in Swedish; (iii) have access to a computer or handheld device with internet access; (iv) currently be employed; (v) and above or below cut-off scores on the following screening questionnaires: ≥14 points on the Perceived Stress Scale; ≤5 points on the Shirom Melamed Burnout Questionnaire (SMBQ); ≤20 points on the Montgomery Åsberg Depression Rating Scale-Self-Rated (MADRS-S); ≤21 points on the Insomnia Severity Index (ISI); and ≤14 points on the Alcohol Use Disorders Identification Test (AUDIT). These cut-off scores were used to include participants with elevated forms of stress and exclude participants with moderate to severe forms of stress, burnout, and other mental health-related symptoms. See section Outcomes for a detailed description of cut-offs for inclusion/exclusion.

In addition, all participants were diagnosed via telephone interviews using the Mini International Neuropsychiatric Interview (Sheehan et al., 1998) and additional criteria from national diagnostic guidelines (Socialstyrelsen, 2003). Participants who did not fulfill a stress-related disorder but were considered eligible according to the inclusion and exclusion criteria presented above were included in the study. Accordingly, mild to moderate forms of DSM Axis-I diagnosis (American Psychiatric Association, 2013) was accepted as co-morbid conditions, as long as they were deemed secondary to the primary adjustment disorder.

Participants were excluded from the study if they: (i) currently were in treatment for stress or burnout; (ii) currently were suffering from bipolar disorder, psychosis, post-traumatic stress disorder (PTSD), eating disorders, substance abuse, severe forms of depression, anxiety disorder or personality disorders; and (iii) were showing suicidal ideation based on Item 9 on the MADRS-S. Participants on medication (e.g., antidepressants or sleep medication) were not excluded from the study but were asked to keep their medication constant during the study period. In total, 156 individuals were screened, and 87 (56 %) were excluded, according to the inclusion and exclusion criteria specified above.

### 2.4 Intervention

The iRTP was based on recovery processes and experiences (psychological detachment, relaxation, mastery, and control), converted into a recovery training intervention, and inspired by Hahn et al. (2011). The iRTP had an emphasis on activities and training of recovery behaviors. In addition to recovery, we also added training on transitional rituals (Ashforth et al., 2000), applied relaxation (Öst, 1987), boundary tactics (Kreiner et al., 2009), along with sleep hygiene and strategies (Åkerstedt et al., 2012; Cheng and Dizon, 2012). The iRTP comprised five modules distributed over five weeks, with modules lasting 60–120 min per week. Each module contained psychoeducation, worksheets, images, cases, audio and video files, and homework assignments. Homework is a key component in psychological treatment, with a clear association between execution and outcomes (Kazantzis et al., 2000). Evidence also suggests that regular support increases participants' adherence and improves the effects of the treatment of stress (Heber et al., 2017). Consequently, each module included both homework and email support (Andersson, 2016). Delayed participants were able to catch up during the final program modules. All participants had access to the intervention one year after the post-treatment assessment. The outline of the iRTP content is depicted in Table 1.

**Table 1:** Outline of the iRTP content.

Module	Name	iRTP content
1	Introduction to recovery training	The first module included an introduction, aim formulation, and psychoeducation about stress and the association between recovery processes and work-life balance. It also focused on the recovery process, and psychological detachment, such as strategies for detachment from stressors at work and in private life (e.g., mindfulness, and detachment-promoting activities). The first module also included transitional rituals (Ashforth et al., 2000), i.e., how to separate work mentally and physically from non-working hours (e.g., having a separate room for work at home).
2	Recovery through relaxation and sleep management	In the second module, participants focused on the recovery processes, relaxation, and sleep management. In this module, they were introduced to a description of the relaxation process and why it is important to recover from stress. Participants were asked to identify relaxing activities and examine commonalities between these activities. The second module also included applied relaxation, a method shown to be effective in reducing tension and stress sensitivity (Öst, 1987), along with a description of the importance of sleep for recovery, including sleep hygiene and strategies (Åkerstedt et al., 2012; Cheng and Dizon, 2012).
3	Mastery and the role of physical exercise in recovery	The third module concentrated on recovery processes mastery and the role of physical exercise in recovery. This module began with a rationale about the concept of mastery and ways to develop and build self-confidence and skills in other areas outside of work. The participants also examined and practiced boundary tactics at work (Kreiner et al., 2009).
4	Recovery in everyday life	The fourth module focused on the recovery processes control and continued training in applied relaxation and boundary-setting tactics. Participants were introduced to the concept of control and why self-determination is important in the recovery from stress. They also were instructed to reflect on what prevents them from spending time on recreational activities and to problem-solve how to take control of their recovery time.
5	Lessons learned, action and relapse plan	In the final module, maintenance, participants focused on summarizing key lessons learned during recovery training, as well as creating an action and relapse plan.

### 2.5 Support

Participants received personalized written messages from a coach with weekly feedback on the exercises. The coaches (four in total) were master's level psychology students in their final year (five). All coaches received weekly supervision from licensed psychologists. The feedback aimed to provide support and encouragement, as well as monitor homework assignments and adherence to the intervention (Andersson, 2016). Treatment-as-usual for health problems was not prohibited, but potentially conflicting treatments (e.g., initiating psychotherapy) were not encouraged. The coaches were asked to minimize their support to one message and 15 min of correspondence per week, per participant.

### 2.6 Primary outcome measure

#### 2.6.1 Recovery Experience Questionnaire

We used the 16-item Recovery Experience Questionnaire (REQ) as the main outcome measure, which includes four factors that represent four different recovery experiences: (i) psychological detachment; (ii) relaxation; (iii) mastery; and (iv) control (Sonnentag and Fritz, 2007). The questionnaire was answered on a five-point Likert scale and has been validated in a Swedish population, showing excellent internal consistency through Cronbach's alpha (α = 0.92) (Almén et al., 2018).

### 2.7 Secondary outcome measures

#### 2.7.1 Shirom-Melamed Burnout Questionnaire

The Shirom-Melamed Burnout Questionnaire (SMBQ; Melamed et al., 1992, Melamed et al., 1999) is a 22-item scale (graded 1–7) used to assess different aspects of chronic stress and burnout (physical fatigue, cognitive weariness, tension, and listlessness). This scale correlates significantly (Grossi et al., 2003) with other well-established questionnaires that measure burnout, e.g., the Maslach Burnout Inventory (Maslach et al., 1997). The SMBQ has an internal consistency reliability of α = 0.92 (Melamed et al., 1999). The cut-off of ≤5 points on the SMBQ was based on the cut-off for severe burnout (Grossi et al., 2003).

#### 2.7.2 Perceived Stress Scale

Perceived stress was measured using a 10-item version of the Perceived Stress Scale (PSS) translated into Swedish (Cohen et al., 1983; Nordin and Nordin, 2013). The PSS-10 is designed to measure the degree to which situations in one's life are appraised as stressful. The Swedish version of PSS has an internal consistency reliability of α = 0.82 and a split-half reliability estimate of 0.84 (Nordin and Nordin, 2013). The cut-off for inclusion in the present trial was based on the mean score (M = 13.96, SD = 6.34) reported in the psychometric evaluation of the Swedish version of the 10-item PSS (Nordin and Nordin, 2013).

#### 2.7.3 Karolinska Exhaustion Disorder Scale

The Karolinska Exhaustion Disorder Scale (KEDS-9) is a nine-item questionnaire that measures symptoms of chronic stress, fatigue, and exhaustion (Beser et al., 2014) using a seven-point scale with a scale range of 0–54. A cut-off score of 19 was shown to discriminate between healthy subjects and patients with chronic stress and exhaustion (Beser et al., 2014). KEDS-9 has satisfactory reliability, at α = 0.94 (Beser et al., 2014).

#### 2.7.4 Montgomery Åsberg Depression Rating Scale

We used the Montgomery Åsberg Depression Rating Scale self-assessment (MADRS-S; Svanborg and Asberg, 1994) to measure symptoms of depression. MADRS-S comprises nine items that measure different symptoms of depression, with each symptom rated on a six-point scale. The instrument has good reliability (Montgomery and Asberg, 1979) and has been validated as an internet-based measure (Holländare et al., 2010; Thorndike et al., 2009). In a comparative study of 10-item (Svanborg and Asberg, 2001), the MADRS-S correlated highly (*r* = 0.87) with the Beck Depression Inventory (Beck et al., 1961), indicating acceptable convergent validity. A cut-off of ≤20 points on the MADRS-S was used according to Montgomery and Asberg, 1979, indicating moderate symptoms of depression.

#### 2.7.5 Generalized Anxiety Disorder Scale

The seven-item Generalized Anxiety Disorder Scale (GAD-7) assesses excessive worry and generalized anxiety disorder. GAD-7 had good internal consistency reliability (α = 0.83), test-retest reliability (*r* = 0.83), and criterion, construct factorial, and procedural validity (Spitzer et al., 2006). A cut-off score of 10 has been suggested to discriminate between healthy subjects and patients with generalized anxiety disorder.

#### 2.7.6 Insomnia Severity Index

Insomnia severity was measured using the Insomnia Severity Index (ISI), a seven-item self-report questionnaire, with total scores ranging from 0 to 28. ISI exhibits adequate internal consistency measures (α = 0.74) and is a sensitive measure for detecting changes in perceived sleep difficulties (Bastien et al., 2001). It previously has been validated for internet use (Thorndike et al., 2011). A cut-off of ≤21 points on the ISI was used, an indication of moderate insomnia severity accruing to Bastien et al. (2001).

#### 2.7.7 International Physical Activity Questionnaire

The International Physical Activity Questionnaire (IPAQ) is a 10-item self-assessment questionnaire (short version) that aims to measure physical activity and inactivity (Craig et al., 2003). Participants are asked to rate all their physical activity (according to intensity level, e.g., walking, moderately strenuous activities, and very strenuous activities) and inactivity (i.e., sedentary time) in minutes over the previous seven days. The IPAQ has demonstrated acceptable reliability, test-retest reliability, and criterion validity (accelerometer) in various contexts and languages (Craig et al., 2003).

#### 2.7.8 Alcohol Use Disorders Identification Test

The Alcohol Use Disorders Identification Test (AUDIT; Saunders et al., 1993) was used to assess potential alcohol dependence or abuse. In a study of the psychometric properties of the Swedish version of AUDIT, both internal and test-retest reliabilities were satisfactory (Selin, 2003). A cut-off of ≤14 points on the AUDIT indicates a risk of overconsumption of alcohol (Berman et al., 2012).

#### 2.7.9 Work Experience Measurement Scale

The Work Experience Measurement Scale (WEMS) gauges the experience of work from a health resource perspective (Nilsson et al., 2013). WEMS comprises 32 items that measure job satisfaction in six different domains (supportive work conditions, internal work experience, autonomy, time experience, management, and process of change) on a six-point scale. Cronbach's alpha on the WEMS has been reported to range from 0.85 to 0.96 (Nilsson et al., 2013).

#### 2.7.10 Work Ability Index

Work Ability Index (WAI) assesses health status and work ability among employees (de Zwart et al., 2002; Eskelinen et al., 1991; Nygård et al., 1991). The WAI comprises different scales, and for the present trial, we used the one-item version, and assessment of the “current work ability compared with the lifetime best” (Ahlstrom et al., 2010). The question is answered on a 10-point scale (0 = completely unable to work; 10 = full work ability at present). Analyses of reliability in the full version of WAI indicate satisfactory internal consistency, with α-levels ranging from 0.79 to 0.80 (Adel et al., 2019; Peralta et al., 2012). A study showed a very strong association between the full WAI and the single-item question and associations with sick leave, health, and symptoms (Ahlstrom et al., 2010).

#### 2.7.11 Brunnsviken Brief Quality of Life Inventory

The Brunnsviken Brief Quality of Life Inventory (BBQ) measures the quality of life within six domains: self-respect; values; leisure time; learning; creativity; and friends. The instrument uses a five-point scale, with a scale range of 0–96. Each domain is assessed according to its importance and contentment. BBQ has satisfactory internal consistency reliability (α = 0.68) and test-retest reliability (*r* = 0.89; Lindner et al., 2016).

#### 2.7.12 Sickness absence

Absence from work was measured using the Trimbos and Institute of Medical Technology Assessment Cost Questionnaire for Psychiatry (TiC-P; Bouwmans et al., 2013). TiC-P has been used in several studies for economic evaluations of healthcare consumption and productivity loss in mental health (Bouwmans et al., 2013). Sickness absences were conceptualized as the self-rated number of days absent from work during the past three months while being physically or mentally ill.

#### 2.7.13 Intervention utility and satisfaction

The participants were asked to rate utility and satisfaction after each module on a 5-point scale (1 = low utility/satisfaction; 5 = high utility/satisfaction).

### 2.8 Statistical analyses

All analyses followed the CONSORT statement for randomized controlled trials (Schulz et al., 2010). Statistical analyses were conducted following the intention-to-treat principle (ITT) using SPSS Version 26 (IBM Corp, Armonk. NY, USA). We used the multiple imputation (MI) procedure to impute missing sum scores for participants who did not complete the post- or six-month follow-up assessments. MI is viewed as a conservative approach for analyzing incomplete data sets, as it takes into account uncertainty due to missing information (Schafer and Graham, 2002). We used all available data from the pre-, post-, and six-month follow-up assessments, as well as age, gender, and education level as predictors. MI is based on the assumption that the data are MAR (missing at random), i.e., that missing data are due to other observed characteristics of the participants. These other variables, e.g., age, gender, and education level, are used for estimating results where values are missing. Means, standard deviations, and effect sizes' standard errors were pooled from five sets of imputations using “Rubin's rules” (Rubin and Schenker, 1991), and the small sample correction for pooled degrees of freedom (Barnard and Rubin, 1999). Formula for Cohen's *d* = (M2 − M1) / SD_pooled_. The group effects on primary and secondary outcome measures of ITT and completers-only data sets were analyzed using analysis of covariance (ANCOVA), with pre-assessment values as covariates. Pooled *F*-values were calculated using RStudio (RStudio Inc., Boston, MA). Cohen's *d* was reported for the between-group effect sizes and corresponding 95 % confidence intervals (95 % CI). Analysis between complete and missing data was analyzed on outcomes at baseline and demographical variables using T- and Chi^2^-tests.

## 3 Results

### 3.1 Participants

Fig. 1 shows the flow of participants, including those who were excluded. After screening and the diagnostic interviews, 87 participants were excluded, mostly because of high scores on one or several of the outcome measures.

### 3.2 Missing data

Baseline data were available for all participants, and the study attrition rate was high in the intervention group: 25.7 % at post-treatment (iRTP = 9 and WLC = 2); 42.9 % at the six-month follow-up (iRTP = 15 and WLC = 3); and 37.1 % at the 12-month follow-up (iRTP = 13) questionnaires. The analysis found no relevant differences between complete and missing data on the baseline outcomes (presented in Table 3) or demographic variables (presented in Table 2).

**Table 2:** Summary of characteristics of study participants.

Characteristics	All participants (N = 69)				iRTP (N = 35)				WLC (N = 34)			
	N	%	M	SD	N	%	M	SD	N	%	M	SD
Socio-demographics												
Age, years			44.4	10.1			44.5	10.7			44.3	9.5
Gender, female	59	85.5			28	80.0			31	91.2		
Married/in a relationship	59	85.5			29	82.9			30	88.2		
Educational level												
Compulsory school												
Upper secondary education	3	8.2			1	6.6			2	15.0		
Higher education	66	95.7			34	97.1			32	94.1		
Working characteristics												
Full-time	57	84.1			29	90.2			28	80.0		
Part-time	12	15.9			6	9.8			6	20.0		
Long-term sick leave	2	2.9			1	2.9			1	2.9		
Hours of overtime, per week			4.2	3.8			4.7	3.9			3.6	3.7
Working sectors^a^												
Social or health	27	39.1			15	42.9			12	35.3		
Education or research	22	31.9			10	28.6			12	35.3		
Communication or IT	9	13.0			5	14.3			4	11.8		
Law, economy or technology	3	4.4			2	5.7			1	1.7		
Others	8	11.6			3	8.6			5	14.7		
Experience												
Previous stress interventions	35	50.7			15	42.9			20	58.8		
First-time help seeker	34	49.3			20	57.1			14	41.2		
Disorders												
F43.2 Adjustment disorder	33	47.8			17	48.6			16	47.1		
F43.8 Exhaustion disorder	0	0			0	0			0	0		
F43.9 Reaction to severe stress.	25	36.2			12	34.3			13	38.2		
F51.0 Non-organic insomnia	17	24.6			12	34.3			5	14.7		
F41.1 Generalised anxiety disorder	3	4.4			2	5.7			1	2.9		
F32.x Depressive episode	3	4.4			2	5.7			1	2.9		
F33.x Recurrent depressive disorder	17	24.6			10	28.6			7	20.6		
F41.0 Panic disorder	2	2.9			1	2.9			1	2.9		
F40.1 Social phobia	4	5.8			3	8.6			1	2.9		
No diagnosis	8	11.6			4	11.4			4	11.8		

Notes: iRTP = Internet-based recovery training program; WL = Waitlist control group.

a Occupations were classified according to the International Standard Classification of Occupations (ISCO).

### 3.3 Baseline characteristics

The study participants' baseline characteristics are presented in Table 2. The sample comprised 69 employees, most of whom were female (85.5 %) with an average age of 44.4 (SD = 10.1). A majority of the participants (71.0 %) were working full-time in the social, healthcare, or educational sectors. 47.8 % fulfilled the ICD-10 diagnosis F43.2 Adjustment disorder and 24.6 % F51.0 Non-organic insomnia. 11.6 % did not meet the criteria for any diagnosis.

### 3.4 Adherence

On average, participants of the iRTP group completed 4.32 modules (SD = 0.80), which equals 86.4 % of the intervention. Regarding dropouts, 14.5 % (iRTP = 9 and WLC = 1) dropped out early (after less than two weeks). The main reason for dropping out was a lack of time. We found no significant differences (all *p*'s between 0.23 and 0.87) between dropouts and complete data on the baseline outcomes or demographic variables (presented in Table 2, Table 3).

**Table 3:** Primary and secondary outcomes for the intention-to-treat sample (iRTP = 35; WLC = 34) at pre, post, six (6FU), and 12-months follow-up (12FU).

Outcome	Group	PRE		POST^n^		6FU^n^		12FU^n^	
		M	SD	M	SD	M	SD	M	SD
Primary outcome									
Recovery (16–80)^a^	iRTP	45.74	8.98	53.23	11.17	54.69	12.31	54.25	9.60
	WLC	46.15	7.02	44.21	8.54	48.67	11.04		
Detachment (4–20)	iRTP	9.00	3.19	12.16	3.41	12.76	3.25	13.50	3.05
	WLC	9.38	3.03	9.91	3.14	11.38	3.83		
Relaxation (4–20)	iRTP	11.77	2.41	13.22	2.42	14.05	2.75	13.55	2.11
	WLC	12.00	2.04	11.94	2.01	12.77	2.49		
Mastery (4–20)	iRTP	12.23	3.88	13.22	3.90	12.84	4.46	12.95	3.44
	WLC	11.65	2.70	9.91	2.82	11.33	3.26		
Control (4–20)	iRTP	12.74	3.39	13.63	3.39	14.00	3.61	14.25	3.40
	WLC	13.12	3.13	12.44	3.48	12.44	3.10		
Health related									
Perceived stress (0–40)^b^	iRTP	21.31	4.15	15.57	5.34	15.30	7.16	15.57	6.20
	WLC	20.91	3.62	18.06	5.09	15.58	5.52		
Burnout (1–7)^c^	iRTP	4.03	0.62	3.24	1.09	3.42	1.37	3.21	0.81
	WLC	4.19	0.74	3.69	0.79	3.40	0.90		
Emotional fatigue	iRTP	4.00	0.84	3.24	1.22	3.22	1.38	3.13	1.01
	WLC	4.11	1.03	3.60	1.08	3.29	1.09		
Cognitive weariness	iRTP	4.08	0.89	3.63	1.21	3.67	1.26	3.61	0.98
	WLC	4.13	0.96	3.91	1.00	3.63	0.99		
Tension	iRTP	4.49	1.14	3.68	1.34	3.70	1.26	3.56	1.15
	WLC	4.54	0.92	4.05	0.91	3.64	1.14		
Listlessness	iRTP	3.76	1.11	3.27	1.55	3.32	1.55	2.81	1.09
	WLC	4.13	0.90	3.31	1.08	3.24	1.20		
Exhaustion (0–54)^d^	iRTP	19.51	5.21	15.86	7.73	14.70	8.20	14.43	6.31
	WLC	19.88	5.56	18.14	6.59	16.38	7.59		
Depression (0–54)^e^	iRTP	12.86	4.98	9.47	5.63	8.90	5.89	8.05	4.08
	WLC	11.50	4.47	11.01	5.46	9.31	5.32		
Anxiety (0−21)^f^	iRTP	6.17	3.36	4.15	2.66	4.01	3.14	3.81	3.30
	WLC	6.21	3.73	5.65	3.43	4.51	3.09		
Insomnia (0–28)^g^	iRTP	9.74	5.53	7.59	5.09	6.53	4.55	6.30	3.29
	WLC	10.38	5.76	9.30	7.09	8.23	6.03		
Alcohol (0–40)^h^	iRTP	2.77	2.11	3.33	2.50	2.26	1.41		
	WLC	3.23	2.40	3.20	3.01	2.97	2.43		
Physical activity^i^	iRTP	2209.44	1725.07	2779.60	1899.02	2284.67	1672.85		
	WLC	2427.13	2379.30	2350.69	1483.90	2293.90	1620.70		
Quality of life (0–96)^j^	iRTP	56.23	19.02	61.33	19.72	59.71	24.21		
	WLC	54.29	18.70	53.37	18.26	56.56	18.30		
Work related									
Work experience (32–192)^k^	iRTP	127.91	24.41	133.07	27.47	127.66	33.80	130.15	29.19
	WLC	128.79	29.50	132.98	25.48	129.09	27.00		
Work ability (0−10)^l^	iRTP	6.86	1.40	7.48	1.05	7.68	1.63	7.50	1.28
	WLC	6.82	1.93	6.87	1.48	7.43	1.36		
Sickness absence^m^	iRTP	2.29	6.34	0.90	3.30	0.80	2.02	0.48	1.25
	WLC	1.85	6.09	1.24	3.72	0.76	3.02		

a REQ = Recovery Experience Questionnaire.

b PSS-10 = Perceived Stress Scale.

c SMBQ = Shirom-Melamed Burnout Questionnaire.

d KEDS=Karolinska Exhaustion Disorder Scale.

e MADRS-S = Montgomery Åsberg Depression Rating Scale-self-assessment.

f GAD-7 = Generalized Anxiety Disorder 7-item scale.

g ISI = Insomnia Severity Index.

h AUDIT = Alcohol Use Disorders Identification Test.

i IPAQ = International Physical Activity Questionnaire (physical activity, total minutes, during the past 7 days).

j BBQ = Brunnsviken Brief Quality of Life Inventory.

k WEMS = Work Experience Measurement Scale;

l WAS = Work Ability Score (item 2 from the Work Ability Index);

m TIC-P = Trimbos and Institute of Medical Technology Assessment Cost Questionnaire for Psychiatry (sickness absence, days, during the past three months according);

n Intention to treat sample. Missing data imputed by multiple imputation.

### 3.5 Primary outcome analyses

As depicted in Table 3, Table 4, the intention-to-treat analysis found that the intervention group improved significantly more than the control group on the primary outcome measure, REQ, at the posttest (*d* = 0.91, 95 % CI 0.38–1.43) and the six-month follow-up (*d* = 0.51, 95 % CI −0.01-1.04). In the following analysis of the recovery experience subscales, participants in the intervention group registered significantly higher scores on psychological detachment (*d* = 0.69, 95 % CI 0.11–1.26), relaxation (*d* = 0.58, 95 % CI 0.10–1.26), and mastery (*d* = 0.97, 95 % CI 0.41–1.54). However, no significant differences were found in the recovery experience control at any time point.

**Table 4:** Results of the ANCOVAs and Cohen's d for the primary and secondary outcome measures (intention-to-treat sample) at post-treatment and six-month follow-up (6FU).

Outcome	POST^a^ Between-groups effects from pre- to posttreatment				6FU^a^ Between-groups effects from posttreatment to follow-up			
	ANCOVA F (3,132)	*P*	Cohen's d	95 % CI	ANCOVA F (3,132)	*P*	Cohen's d	95 % CI
Primary outcome								
Recovery (16–80)^b^	16.45	<0.000	0.91	0.38–1.43	5.45	0.021	0.51	−0.01–1.04
Detachment (4–20)	7.53	0.010	0.69	0.11–1.26	2.24	0.131	0.39	−0.19–0.96
Relaxation (4–20)	4.08	0.029	0.58	0.10–1.26	2.90	0.124	0.49	−0.17–1.15
Mastery (4–20)	17.46	<0.000	0.97	0.41–1.54	3.18	0.040	0.39	−0.12–0.90
Control (4–20)	3.02	0.086	0.35	−0.16–0.86	1.25	0.177	0.26	−0.25–0.76
Health related								
Perceived stress (0–40)^c^	3.20	0.047	0.48	0.10–1.06	0.08	0.862	0.03	−0.48–0.54
Burnout (1–7)^d^	2.60	0.284	0.47	0.14–1.09	0.17	0.527	0.02	−0.55–0.58
Emotional fatigue	1.57	0.287	0.31	−0.20–0.82	0.00	0.824	0.06	−0.58–0.70
Cognitive weariness	0.96	0.328	0.25	−0.26–0.76	0.17	0.844	0.04	−0.50–0.57
Tension	1.90	0.405	0.33	−0.20–0.86	0.21	0.687	0.04	−0.51–0.60
Listlessness	0.42	0.539	0.03	−0.47–0.54	0.20	0.642	0.05	−0.47–0.57
Exhaustion (0–54)^e^	1.83	0.182	0.32	−0.17–0.81	0.51	0.509	0.21	−0.35–0.78
Depression (0–54)^f^	2.77	0.107	0.28	0.22–0.78	0.56	0.601	0.08	−0.52–0.68
Anxiety (0–21)^g^	3.89	0.041	0.49	−0.03–1.02	0.39	0.666	0.17	−0.52–0.86
Insomnia (0–28)^h^	1.12	0.278	0.28	0.21–0.77	1.27	0.227	0.32	−0.17–0.82
Alcohol (0–40)^i^	0.01	0.441	0.04	−0.54–0.45	0.70	0.417	0.34	−0.26–0.93
Physical activity^j^	1.09	0.317	0.25	−0.28–0.78	0.30	0.606	0.02	−0.59–0.64
Quality of life (0–96)^k^	3.13	0.048	0.47	0.02–0.96	0.30	0.539	0.15	−0.40–0.70
Work related								
Work experience (32–192)^l^	0.15	0.640	0.00	−0.53–0.53	0.50	0.502	0.04	−0.64–0.55
Work ability (0–10)^m^	3.84	0.038	0.47	−0.08–1.01	0.47	0.806	0.17	−0.42–0.76
Sickness absence^n^	0.20	0.680	0.09	−0.43–0.62	1.42	0.239	0.02	−0.56–0.59

a Intention to treat sample. Missing data imputed by multiple imputation.

b REQ = Recovery Experience Questionnaire.

c PSS-10 = Perceived Stress Scale.

d SMBQ = Shirom-Melamed Burnout Questionnaire.

e KEDS=Karolinska Exhaustion Disorder Scale.

f MADRS-S = Montgomery Åsberg Depression Rating Scale-self-assessment.

g GAD-7 = Generalized Anxiety Disorder 7-item scale.

h ISI = Insomnia Severity Index.

i AUDIT = Alcohol Use Disorders Identification Test.

j IPAQ = International Physical Activity Questionnaire (physical activity, total minutes, during the past 7 days).

k BBQ = Brunnsviken Brief Quality of Life Inventory.

l WEMS = Work Experience Measurement Scale.

m WAS = Work Ability Score (item 2 from the Work Ability Index).

n TIC-P = Trimbos and Institute of Medical Technology Assessment Cost Questionnaire for Psychiatry (sickness absence, days, during the past three months according).

### 3.6 Secondary outcome analyses

Table 4 presents the results from the intention-to-treat analysis of the secondary outcome measures. Compared with the controls, participants in the intervention group reported significant and small to moderate effects on perceived stress (*d* = 0.48), anxiety (*d* = 0.49), quality of life (*d* = 0.47), and work ability (*d* = 0.23) at post-assessment. Both the iRTP and WLC groups continued to improve during the six- and 12-month follow-up periods, but no significant differences were found at any time point regarding burnout, exhaustion, depression, physical exercise, work experience, or sickness absences.

### 3.7 Client satisfaction and intervention support

Client utility and satisfaction with the iRTP program were assessed on a five-point scale (1 = low satisfaction; 5 = high satisfaction). The utility was given an average of 4.33 (SD = 0.75) and satisfaction, of 4.32 (SD = 0.69). Only one participant was hesitant about whether he or she would recommend the program. 24 % of the participant in the iRPT group perceived the content as extensive and would prefer more time to complete and reflect on each module. The most appreciated modules were psychological detachment, relaxation, boundary tactics, physical exercise, and work/life balance. Less appreciated or essential modules were physical exercise (expressed by those already exercising) and example persons (could not identify with the person). Additionally, 92 % experienced the support as relevant and helpful. However, 16 % of participants would have preferred continuous monitoring and dialogue with their coach.

### 3.8 Long-term follow-up and maintenance of gains

Participants in the iRTP group sustained the initially gained improvement on all outcomes from post-treatment to the six- and 12-month assessments. However, the only outcomes with significant between-group effects were recovery experiences, relaxation (d = 0.49), and mastery (d = 0.39). The long-term follow-up results were based on the intention-to-treat sample.

### 3.9 Completers-only analyses

Complete case analysis revealed significant (*p* < 0.001) and large effects for the primary outcome, REQ, at post-assessment (*d* = 1.03, 95 % CI 0.47–1.59) and moderate effects at the six-month follow-up (*d* = 0.67, 95 % CI 0.08–1.26). Significant (*p* < 0.01) and small to moderate and significant effects also were found in the completers' analyses of several of the secondary outcomes (e.g., PSS *d* = 0.57, 95 % CI 0.03–1.11).

## 4 Discussion

In this randomized, controlled pilot trial, we examined the efficacy of a brief internet-based recovery training program targeting a clinical sample of distressed employees. We expected that the intervention group would improve significantly on the primary outcome REQ compared with a waitlist control condition. In addition, we examined whether participants in the intervention group would improve on several other health (perceived stress, burnout, exhaustion, depression, anxiety, physical exercise, and alcohol consumption) and work-related (work experience, work ability, and sickness absences) outcomes.

The results confirmed our primary assumption that the intervention was effective in improving the main outcome, REQ, including subscales, psychological detachment, relaxation, and mastery. However, no significant differences were found between groups on the REQ subscale control. Secondary explorative analyses indicated positive effects on perceived stress, anxiety, quality of life, and work ability, but contrary to our expectations, no significant differences were found at any time point regarding burnout, exhaustion, depression, insomnia, alcohol consumption, physical exercise, work experience, or sickness absences. As predicted, the results on the primary and secondary outcomes remained stable in the intervention group at the six- and 12-month follow-ups.

The finding that recovery intervention leads to increases in recovery outcomes corresponds with previous trials and reviews (Almén et al., 2020; Hahn et al., 2011; Karabinski et al., 2021; Thiart et al., 2015). In the present trial, mastery gained the greatest effect sizes (*d* = 0.97), followed by psychological detachment (*d* = 0.69), and relaxation (*d* = 0.58). Psychological detachment followed by relaxation is usually regarded as the most consistent aspect of recovery (Sonnentag et al., 2017). In the present trial, the RTP group exhibited larger between-group effect sizes on psychological detachment from work compared to the average effects (*d* = 0.38) reported in the meta-analysis by Karabinski et al. (2021). However, the meta-analysis did include studies with both between and within-group designs (without a control group), which limits the possibility of valid comparison. Yet, similar effects were found in a study by Thiart et al. (2015) on an internet-based, six-week, sleep and recovery program that targeted teachers with insomnia. Compared to the study by Thiart et al. (2015), the between-group differences declined at the six month follow-up on the primary and secondary outcomes. Perhaps these differences could be explained by spontaneous remission in the control group, which is common in stress and adjustment disorders (Lorenz et al., 2018).

No significant effect was found on the REQ subscale control. This finding might be due to the fairly small sample size and insufficient statistical power. Complete case analysis of the present trial showed significant (*p* < 0.05) and moderate effect sizes on the recovery experience control (*d* = 0.54, CI 95 % 0.01–1.08).

Results on the secondary outcomes on perceived stress, anxiety, and quality of life were comparable with the effects found in a meta-analysis (Heber et al., 2017) on internet-based interventions for stress (e.g., perceived stress, *d* = 0.43, and anxiety, *d* = 0.32). Interestingly, we found positive effects on work ability. However, the effect was small (*d* = 0.47), but comparable to effects found in a recent meta-analysis (Finnes et al., 2019) on the effects of psychological interventions on sickness absences due to common mental disorders (Hedges' *g* = 0.22). These results are promising, considering that low work ability and work participation have been associated with higher risks for long-term sick leave, poorer well-being, and high personal and societal costs (Finnes et al., 2019; Hassard et al., 2018).

In line with the prior quasi-experimental evaluation by Hahn et al. (2011), no significant effects were found at any time point regarding burnout, exhaustion, depression, insomnia, physical exercise, alcohol consumption, work experience, or sickness absences. The insignificant results on depression, alcohol consumption, work experience, and sick leave can be explained by the fact that the main focus of the present trial was to evaluate the effects of a recovery program on recovery and stress-related outcomes. We did not include any specific interventions or techniques, neither directly nor indirectly, addressing these areas or outcomes. In the case of burnout, exhaustion, and insomnia, we expected that the focus on recovery processes, relaxation, sleep management, and physical exercise would reduce some of the negative effects of burnout, exhaustion, and insomnia. It is possible that brief interventions, like the present with a timeframe of five weeks, are too short to generate effects on disorders such as burnout, exhaustion, and insomnia. Another plausible explanation is that we did not include relevant components, which would have affected these outcomes. For example, we did not include any behavioral activation or sleep restrictions, which have been shown to be effective in reducing depression and insomnia, respectively (Cheng and Dizon, 2012; Martell et al., 2001; Spanhel et al., 2022). In line with this reasoning, we found significant and large effects on burnout, depression, and insomnia in a recent study (Persson Asplund et al., 2023) of a teen-week iCBT for stress-related disorders, including both behavioral activation and sleep restriction.

### 4.1 Limitations

This study also has limitations. First, given the timeframe and conditions set by the research grant, we did not manage to recruit >69 participants. These circumstances may have resulted in weak statistical power and the risk of Type I and II errors (especially in the case of samples with high attrition). Thus, future studies would benefit from a larger sample size to reduce the risk of bias. Another consideration regarding power is the correlation between sample size and effect size, with larger effects in small samples (Kühberger et al., 2014). Second, there was substantial attrition in the intervention group: 25.7 % at post-treatment; 42.9 % at the six-month follow-up; and 37.1 % at the 12-month follow-up. According to guidelines, the limits of the acceptable drop-out rate are 20 % for short-term and 30 % for long-term follow-ups (Furlan et al., 2009). Although we applied Multiple imputation, which is considered a conservative approach to handle missing data (Schafer and Graham, 2002), and even though attrition is common in internet-based intervention studies (Eysenbach, 2005), we cannot rule out a potential bias caused from missing data. Further, the extensive inclusion of outcome measures may have implied a great burden for the participants, resulting in a higher attrition rate. Third, we used an open recruitment strategy, with the potential risk of selection bias. For example, 92 % of the participants had a university-level education background, compared with 28 % in the general population (Statistics Sweden, 2020), and 62 % were working in the social, healthcare, or educational sectors. Therefore, future studies are needed that comprise participants who are more representative of the general working population. Perhaps integrating iRTP into the workplace could lower thresholds and offer a successful approach by including various employees from different industries. Fourth, we did not include any mediator or moderator analyses in this controlled pilot trial, which, again, is related to the fairly small number of participants. Thus, future studies could be designed with repeated assessments to test for mediating and moderating mechanisms. For instance, it would be interesting to examine the mediating role of recovery activities/behaviors and if the change in activities could lead to effects on recovery experiences and even have a preventive potential for long-term mental health consequences, such as burnout, depression, and insomnia. For instance, both psychological detachment and sleep management might have the potential to reduce work-related rumination and impaired sleep, antecedents to burnout, depression, and insomnia (Grossi et al., 2015; Vahle-Hinz et al., 2014). Fifth, it is possible that the short-term effects were underestimated as the post-assessment was conducted directly after five weeks. If participants would have given some time to absorb and apply the last module, they would perhaps benefit more and could enfold the full effect of the RTP. This procedure was applied in the study by Ebert et al. (2015) and could be a model for future studies.

### 4.2 Conclusions and future remarks

In conclusion, the preliminary results suggest that employees from various professions could learn how to recover from elevated symptoms of stress via a brief internet-based recovery training intervention. This study also indicates that the initially gained improvements could be sustained over time. This type of accessible and brief recovery intervention could potentially prevent and reduce the negative effects of stress, as well as improve recovery and quality of life. However, more research is needed with larger samples before further conclusions can be drawn. Future studies could bring new insight into the mechanisms of change, the efficacy of various recovery processes, and the role of RTPs within the organizational context.

## Declaration of competing interest

The authors declare that they have no known competing financial interests or personal relationships that could have appeared to influence the work reported in this paper.
